# A School-Level Examination of the Association between Programs and Policies and Physical Activity Outcomes among Females from the COMPASS Study

**DOI:** 10.3390/ijerph18063314

**Published:** 2021-03-23

**Authors:** Kathleen E. Burns, Julianne Vermeer, Kate Battista, Scott T. Leatherdale

**Affiliations:** School of Public Health and Health Systems, University of Waterloo, Waterloo, ON N2L 3G1, Canada; jvermeer@uwaterloo.ca (J.V.); kbattista@uwaterloo.ca (K.B.); sleatherdale@uwaterloo.ca (S.T.L.)

**Keywords:** youth, school, physical activity, programs, policies, female

## Abstract

(1) The majority of Canadian youth are not meeting physical activity guidelines, and more female than male youth are falling short of these recommendations. School programs and policies are a viable strategy to improve youth physical activity. However, they may differentially affect female and male activity. This study aimed to examine school-level differences in physical activity outcomes among male and female students and to explore how school programs and policies associate with school-level physical activity outcomes among females. (2) This study used data from 136 schools participating in year 7 (Y7 2018–2019) of the COMPASS study. Data on school programs and policies and on student physical activity were collected. School-level means and percentages for outcomes were calculated and compared between males and females and the impact of physical activity programs and policies on female physical activity outcomes were examined. (3) More males met the guidelines, achieved more strength training days and physical activity minutes compared to females. The number of female varsity sports, community partnerships and fitness ambassadors were all positively and significantly associated with female physical activity. (4) Supportive physical activity environments fostered by offering varsity sports, establishing community partnerships and positive role models may promote physical activity among female youth.

## 1. Introduction

Physical activity is important for youth as it aids in healthy development and disease prevention later in life [1,2,3]. Physical activity is also positively associated with academic performance [4,5] and may help to reduce symptoms of depression and anxiety in youth [1,2]. For optimal health, youth should accumulate sufficient levels of physical activity, as outlined in the Canadian 24 h Movement Guidelines developed by the Canadian Society for Exercise Physiology (CSEP). The CSEP “Sweat” guidelines recommend that youth aged 5–17 years accumulate at least 60 min of daily moderate-to-vigorous physical activity (MVPA) involving a variety of aerobic activities, as well as vigorous physical activity and muscle and bone strengthening activities at least 3 days per week [6]. Despite the benefits of physical activity and the introduction of the CSEP guidelines in 2016, many Canadian youth fall short of meeting physical activity recommendations. Previous research found that only approximately 35% of Canadian youth aged 5–17 years of age met the recommendation of at least 60 min of MVPA and at least 3 days of strength training [7,8,9]. Additionally, male youth are more likely to meet these activity guidelines [7,9] and are more physically active compared to female youth [7,8,9]. This is concerning, as physical inactivity is the leading behavioural risk factor for death and life expectancy lost in Canadian females [10]. Not only are Canadian youth not meeting the physical activity recommendations, but physical activity decreases over time in this age group [11,12,13,14,15,16,17], highlighting the importance of physical activity interventions among this population. Youth physical inactivity is not unique to the Canadian context, as globally, the majority of youth are not meeting the current physical activity recommendations of at least one-hour per day [18]. Increasing physical activity among youth should be a public health priority to address physical inactivity [7,8,9] and reduce the population-level health burden in the future [19].

Ecological models suggest that physical activity is influenced by the surrounding environment in combination with individual factors [20]. The school is an important environment influencing youth physical activity because school-based programs, policies and facilities interrelate to impact youth physical activity behaviour [21,22,23]. Comprehensive strategies to address youth physical inactivity should incorporate creating and promoting supportive school physical activity environments [24,25,26], as youth spend a large proportion of time at school [27]. Previous research has found school-level characteristics to be associated with higher levels of youth physical activity compared to individual factors [28,29], and the effect of these school-level factors on physical activity are significantly different between males and females. For example, although intramural and varsity sports are positively associated with physical activity [30,31,32,33,34], these school-based sport programs affect male and female physical activity differently because males are more likely to participate in school-based sports [35,36] and participate in higher intensity physical activity during these activities compared to females [37]. Examples of school-level physical activity policies that are positively associated with physical activity include offering access to school physical activity facilities [38], providing access to after-school supervised activity [34], creating community partnerships to improve access to community health facilities [23], and encouraging active transportation [39]. However, there may be differences in how such policies associate with physical activity between males and females [40], and this relationship is not well understood. For example, previous research has shown school physical activity environments (e.g., open areas, gymnasiums) to be correlated with higher levels of physical activity among males but not females [41], suggesting that the effectiveness of policies increasing access to these activity settings may be different for males and females. These differences between male and female youth on the association between the school environment and physical activity may be explained by the different motivations and barriers to physical activity participation. For instance, male youth are more likely to report enjoyment, competition, becoming stronger and winning as motivations for physical activity, and female youth are more likely to report peer socialization, inclusion and keeping fit as motivations for physical activity [42,43,44]. Additionally, barriers to physical activity such as perceived lack of competence, lack of time, access to facilities, and dislike of highly structured activities, may be more problematic for female youth compared to male youth, further explaining the difference in these associations [40]. Considering that female youth are an at-risk group for physical inactivity, it is important to determine if school-level programs and policies are positively associated with female physical activity.

Previous research has examined the impact of school-based programs and policies on youth physical activity outcomes at the individual level [21,23,41,45,46]. However, limited research has been performed to examine how school-level physical activity programs and policies impact physical activity outcomes at the school level. Examining the effect of these programs and policies at the school level, as opposed to the individual level, provides data about the effectiveness at the population level. These population-level data are useful for decision making, resource allocation and examining how physical activity programs and policies impact the school population as a whole, as opposed to how it may benefit the average student [47].

Considering the lack of school-level research examining the effect of physical activity programs and policies on population-level female physical activity, this paper aims to examine how school-based programs and policies affect school-level physical activity outcomes among females as outlined in the CSEP guidelines. More specifically, the objectives of this research are to: (1) examine school-level differences between male and female youth on (i) percentage meeting the CSEP guidelines, (ii) weekly days of strength training, and (iii) average daily MVPA minutes. If there are school-level differences between male and female physical activity outcomes, then a secondary objective is to (2) examine how school-level policies and programs are associated with these school-level physical activity outcomes among female students.

## 2. Materials and Methods

### 2.1. Procedures and Participants

The COMPASS study is a prospective cohort study that collects hierarchical (school- and student-level) longitudinal data from a convenience sample of Canadian secondary schools and students from Alberta, British Columbia, Ontario and Quebec. School-level data are collected on programs, policies and the built environment, and student-level data are collected on several health outcomes including physical activity, sedentary behaviour, diet, substance use and mental health. This study utilized cross-sectional school-level and student-level data from Year 7 (Y7: 2018–2019) of the COMPASS study. The Y7 sample includes 74,501 students from 136 schools in Ontario (*n* = 61), Alberta (*n* = 8), British Columbia (*n* = 15) and Quebec (*n* = 52). These students included in the sample had a mean age of 15 years (SD = 1.5 years) at the time of data collection. COMPASS uses an active information passive-consent protocol which is advantageous in self-report research to produce results that reduces self-selection and response biases [48,49,50]. A complete description of the COMPASS study can be found online (www.compass.uwaterloo.ca, accessed on 17 March 2020).

### 2.2. Measures

#### 2.2.1. School-Level Programs and Policies Data

Data on physical activity programs and policies are collected using the School Programs and Policies Questionnaire (SPP). The SPP is an online questionnaire completed by a school contact that is familiar with the school’s programs and policies. Specific questions were examined within the SPP to determine the types and numbers of physical activity programs and to determine the physical activity policies that were implemented at the time of data collection.

##### Physical Activity Programs

*Number of Intramural Sports:* School intramural programs were measured by asking schools to indicate which intramural programs/club activities were offered in the past 12 months from a list of 19 common intramurals, with options to write in additional intramurals not on the list. Separate response options were given to indicate program availability for “Girls Only”, “Boys Only”, and “Co-ed”. The number of intramurals for each group were counted as continuous variables.

*Number of Varsity Sports:* School varsity programs were measured by asking schools to indicate which interschool or varsity programs were offered in the past 12 months from a list of 22 common varsity sports, with options to write in additional sports not on the list. Separate response options were given to indicate program availability for “Junior Girls”, “Senior Girls”, “Junior Boys” and “Senior Boys”. The number of varsity sports for girls and boys (junior and senior combined) were counted as continuous variables.

##### Physical Activity Policies

*Partnerships:* Schools were asked about the nature of any partnerships with external health and fitness organizations (e.g., YMCA, GoodLife) over the past 12 months using three response selections: (1) “access to off-site fitness facilities for school-related activities”, (2) “reduced cost of student memberships negotiated by the school”, and (3) “fitness ambassadors (individuals that promote physical activities among students) working in the school”.

*Access to indoor and outdoor facilities during school time:* Additional school policies regarding access to indoor and outdoor physical activity facilities were assessed by asking “Do the majority of students at your school have regular access to INDOOR [OUTDOOR] physical activity areas during non-instructional school time?”. Responses included options for facilities on and off school grounds and were dichotomized as yes/no.

*Access to equipment during school time:* Equipment access during non-instructional time was also assessed by asking “Do students have access to physical activity equipment such as soccer and basketballs during non-instructional times throughout the school day?”. Responses were dichotomized into “Always” and “Sometimes” or “Never”.

*Access to indoor and outdoor facilities outside of school time:* Policies regarding access to facilities outside of school hours was assessed by asking “Outside of school hours, does your school permit regular student access to the following? (Check all that apply)” Multiple time periods for “Before School”, “After School”, “Evenings” and “weekends” could be selected and separate responses were permitted for indoor and outdoor facilities. Responses were dichotomized to “Yes” if any were selected and “No” otherwise.

*Access to equipment outside of school time:* Equipment access outside of school time was assessed by asking “Outside of school hours, does your school permit regular student access to equipment (e.g., soccer balls and basketballs)? (Check all that apply)” Multiple time periods for “Before School”, “After School”, “Evenings” and “weekends” could be selected and responses were dichotomized to “Yes” if any were selected and “No” otherwise.

##### Demographic Data

School-level data on urbanicity and school-area median income (a measure of socioeconomic status) were collected from the 2016 Canadian census [51]. Data on school enrolment and province were recorded at the time of the data collection.

##### Student-Level Physical Activity Outcomes

Student-level data are collected using the COMPASS Questionnaire (Cq), an anonymous, self-administered, paper-based questionnaire. The Cq is completed by students in-class and takes approximately 40 min to complete. The response rate in year 7 was 84.2%, with the primary reason for non-response being absenteeism or scheduled spare at the time of the data collection.

*Meeting CSEP Guidelines:* To determine the percentage of students meeting the 24 h Movement Guidelines implemented by CSEP [6], a variable was derived from student’s responses to MVPA and days of strength training questions. Students who completed at least 60 min of average daily MVPA as well as completed 3 of more days of strength training were classified as meeting the CSEP guidelines.

*Weekly Days of Strength Training:* To examine weekly days of strength training, students are asked “On how many days in the last 7 days did you do exercises to strengthen or tone your muscles? (e.g., push-ups, sit-ups, or weight-training)”, with the response options of “0 days”, “1 day”, “2 days”, “3 days”, “4 days”, “5 days”, “6 days” and “7 days”.

*Average Daily MVPA:* Average daily MVPA in minutes was derived from examining students’ responses to questions about moderate and vigorous physical activity. First, students are asked to state the number of minutes of moderate and vigorous physical activity they participated in for the past 7 days (Monday–Sunday). Second, the total combined moderate and hard physical activity is then calculated for each day, and thirdly, the sum of MVPA for each day is then divided by 7 days to calculate the average combined MVPA per day. This self-reported measure of MVPA has acceptable reliability (ICC = 0.75) and validity for use in research involving school-age youth [52,53].

### 2.3. Analysis

School-level averages for minutes of average daily MVPA and days of strength training, as well as the percentage of students meeting physical activity guidelines were calculated for males and females. 

Sample statistics were used to describe the sociodemographic characteristics of the school-level sample. Paired *t*-tests were used to examine the differences between male and females on: (i) percentage meeting the CSEP guidelines, (ii) weekly days of strength training, and (iii) average daily MVPA minutes. Pearson correlation tests were used to examine the association between the number of physical activity programs and: (i) percentage meeting the CSEP guidelines, (ii) weekly days of strength training, and (iii) average daily MVPA minutes among female students. Lastly, one-way analysis of variable (ANOVA) was used to examine the associations between school-level policies on: (i) percentage meeting the CSEP guidelines, (ii) weekly days of strength training, and (iii) average daily MVPA minutes among female youth. All bivariate calculations were run separately on each predictor variable. SAS 9.4 (SAS Institute, Cary, NC, USA)) was used for all analyses.

## 3. Results

### 3.1. Sample Characteristics

Table A1 (see Appendix A) includes a sociodemographic description of the 136 schools that were analyzed in this sample. By province, 5.9% were from Alberta, 11.0% from British Columbia, 44.9% from Ontario and 38.2% from Quebec. The majority of schools (47.1%) were classified as small urban/rural, while 42.6% were considered large urban and 10.3% were medium urban. Schools were close to evenly distributed across the income categories, with 25.7% classified as less than $50,000, 24.3% classified as $50,000 to $75,000, 26.5% classified as $75,000 to $100,000 and 23.5% classified as over $100,000. The majority of schools had a student population of between 501 and 950 (40.4%), with the smallest percentage of schools having a student population greater than 951 (11.7%).

### 3.2. **Objective 1.** School-Level Differences between Male and Female Youth on Physical Activity Outcomes

Table 1 shows the school-level physical activity outcomes by gender. There were significant differences on all female and male physical activity outcomes, as a larger percentage of males met the CSEP guidelines and had higher average weekly strength training days and higher average daily MVPA compared to females.

**Figure 1:**
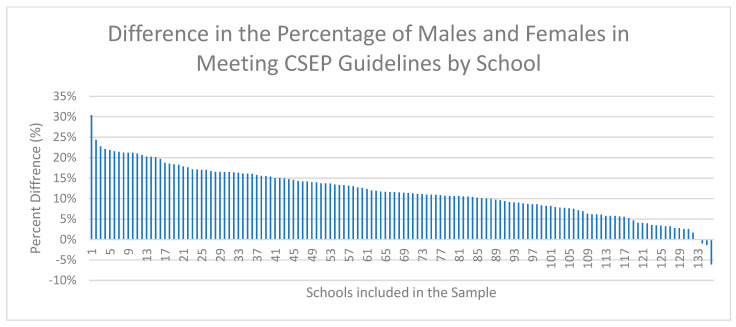
School-level differences between the percent of males and the percent of females meeting the CSEP guidelines from Year 7 (2018–2019) of the COMPASS study. Note: The difference is calculated as the percentage of males meeting guidelines minus the percentage of females meeting guidelines.

**Figure 2:**
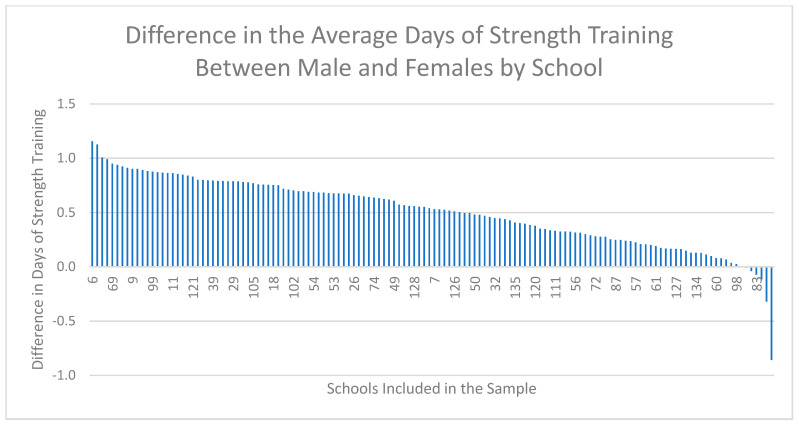
School-level differences (*n* = 136) between the average days of strength training per week for male and female students from Year 7 (2018–2019) of the COMPASS study. Note: The difference is calculated as the average weekly strength training days for males minus the average weekly strength training days for females.

**Figure 3:**
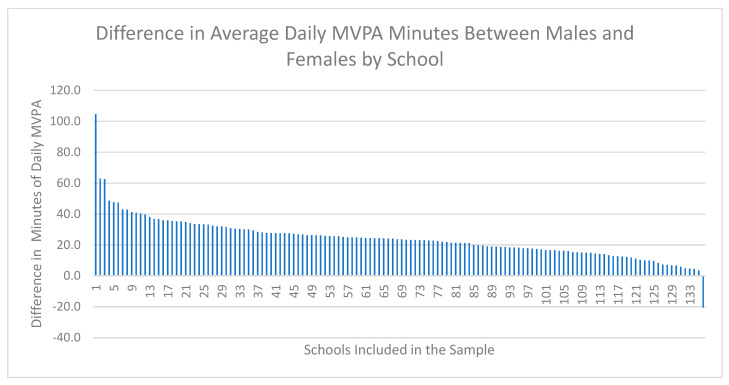
School-level differences between average daily minutes of MVPA for male and female students from Year 7 (2018–2019) of the COMPASS Study. Note: The difference is calculated as the average daily minutes of MVPA for males minus the average daily minutes of MVPA for females.

Figure 1, Figure 2 and Figure 3 demonstrate the range of school-level differences between male and female physical activity outcomes. There was variability between schools in the magnitude differences between male and female students on the: (1) percentage of students meeting CSEP guidelines, (2) average days of strength training and (3) average daily minutes of MVPA.

#### 3.2.1. Outcome 1: Meeting CSEP’s 24 h Movement Guidelines

Figure 1 visually presents the difference between the percentage of males and the percentage of females meeting the CSEP guidelines for each school included in the sample. Schools with a positive percentage indicate that a greater percentage of male students compared to females met the CSEP guidelines, and schools with a negative percentage indicate that a greater percentage of females compared to males met the CSEP guidelines. The purpose of this figure is to demonstrate that the majority of schools had a higher percentage of males, compared to females, meeting the CSEP guidelines. More specifically, the percentage of males meeting the CSEP guidelines was higher compared to females in 97% of schools. One school has an equal percentage of males and females meeting the CSEP guidelines, and 3 schools had a larger percentage of females meeting the CSEP guidelines compared to males (range = −6% to 30%).

#### 3.2.2. Outcome 2: Average Days of Strength Training

Figure 2 visually presents the difference between males and females on the average number of strength training days per week for each school included in the sample. Schools with a positive number of average strength training days indicates males achieved a higher number of average strength training days per week compared to females, and schools with a negative number of average strength training days indicates females achieved a higher number of average strength training days per week compared to males. The purpose of this figure is to demonstrate that the days of strength training were higher for males compared to females in the majority of schools. More specifically, days of strength training were higher for males compared to females in most schools, and the difference between males and females on weekly days of strength training ranged from 0.9 to 1.2 days. Strength training was equivalent between males and females in five schools, while females accumulated more average days of strength training compared to males in four schools. The largest difference in average days of strength training was 1.2, where males completed 1.2 more days of strength training days per week compared to females.

#### 3.2.3. Outcome 3: Average Daily Minutes of MVPA

Figure 3 visually presents the difference between males and females on the average daily minutes of MVPA for each school included in the sample. Schools with a positive number of average daily MVPA minutes indicates that males achieved more average daily MVPA minutes compared to females, and schools with a negative number of average daily MVPA minutes indicates that females achieved more average daily MVPA minutes compared to males. The purpose of this figure is to demonstrate that the average daily minutes of MVPA were higher for males compared to females in the majority of schools. More specifically, males achieved more MVPA compared to females in all schools with the exception of one school, where females completed on average 20.6 more minutes of MVPA compared to males. For all other schools, males achieved more daily MVPA minutes compared to females, with a range of −20.6 to 104.6. The largest difference in average daily MVPA was 104.6 min, where males completed 104.6 more average minutes of MVPA compared to females.

### 3.3. **Objective 2.** Association between School-Level Programs and Policies on School-Level Physical Activity Outcomes among Female Students

#### 3.3.1. School-Level Programs and Physical Activity Outcomes:

Table 2 shows the associations between school-level programs offered at school and physical activity outcomes for female students. Schools with greater number of female varsity teams had a significantly higher percentage of female students meeting the CSEP guidelines (*p* < 0.0001), higher average days of strength training among female students (*p* < 0.0001) and higher average MVPA among their female students (*p* < 0.0001), all compared to schools with lower numbers of female varsity teams. The number or type of female only intramural programs had no impact on any of the outcomes.

#### 3.3.2. School-Level Policies and Physical Activity Outcomes:

Table 3 shows the associations between school-level policies and female physical activity outcomes. There was a significantly higher percentage of female students meeting the CSEP guidelines (35.2%, *p* = 0.0476) and there was a higher average MVPA among females (103.7 min, *p* = 0.0417) in schools that partnered with external facilities to provide student memberships at a reduced cost to these facilities, compared to schools without such partnership. Schools that partnered with fitness ambassadors showed a significantly higher percentage of females (38.4%, *p* = 0.0004) meeting the CSEP guidelines, higher average days of strength training (2.4 days, *p* = 0.0062) and higher average MVPA (113.3 min, *p* < 0.0001), all compared to schools with no fitness ambassador. There were no significant differences in the outcomes between female students in schools partnering to provide students access to external facilities compared to female students attending schools without such partnerships.

Schools that permitted access to indoor facilities during non-instructional school time had significantly lower percentages of females meeting the CSEP guidelines (31.2%, *p* = 0.0017), fewer average number of strength training days (2.2 days, *p* = 0.0266) and lower average MVPA (95.2 min, *p* = 0.0011), all compared to schools without access to indoor facilities during non-instructional time. Schools that permitted access to equipment during non-instructional time had fewer female students meeting the CSEP guidelines (29.9%, *p* = 0.0178) and lower average MVPA (91.6 min, *p* = 0.0051), all compared to schools with no access to equipment during non-instructional time. There were no significant differences in the outcomes between females attending schools that permit access to outdoor facilities during non-instructional time compared to females attending schools without such permissions.

Schools that permit access to their indoor facilities outside of school hours had significantly lower female average daily MVPA (95.3 min, *p* = 0.0135), compared to schools without this access. Schools that permitted access to equipment during outside of school hours had lower percentages of female students meeting the CSEP guidelines (30.7%, *p* = 0.0218) and lower female average MVPA (94.6 min, *p* = 0.0468), all compared to schools without these permissions.

## 4. Discussion

This study addressed an important gap in the literature by examining how school-level physical activity programs and policies associate with school-level physical activity outcomes among female secondary school students. More specifically, we utilized a large cross-sectional sample of Canadian secondary school students to examine school-level differences between male and female students on physical activity outcomes and subsequently examined how school-level programs and policies are associated with key outcomes of the CSEP guidelines; the percentage of students meeting the CSEP guidelines, the average number of weekly strength training days and average daily MVPA, among female secondary school students. Our results consistently showed that between schools, a larger percentage of males met the CSEP guidelines and had higher average weekly strength training days and higher average daily MVPA compared to females. Additionally, we found that specific school-based programs and policies were positively associated with female physical activity which is important when considering school-level strategies to promote female physical activity.

Compared to females, a larger proportion of males met the CSEP guidelines, and males consistently engaged in more weekly days of strength training and more minutes of daily MVPA compared to their female counterparts. This finding is consistent with other research, as males are more physically active compared to females on average [9,12,17,37,54]. While a gender gap is evident, this study reveals that schools participating in the COMPASS study display varying degrees of gender differences in physical activity outcomes. Our results also demonstrate that not all school-level programs and policies may be effective strategies for increasing physical activity among female youth, as some policies (community fitness center partnerships, fitness ambassador) and programs (varsity sports) were positively associated with physical activity outcomes, while access to school fitness equipment and facilities were not.

The relationship between physical activity and youth has been frequently described using the socio-ecological model in the literature, where intrinsic/personal factors, interpersonal (family and friends), built environment and social/policy factors interrelate to influence physical activity [29,55,56]. More specifically, female physical activity may more likely be attributed to personal factors and the influence of family and friends, and less likely related to factors related to the built environment, such as school facilities [55]. This was reflected in our study results which found that access to school equipment and facilities and number of intramural programs were not positively associated with physical activity, and in fact a counter-intuitive negative correlation was observed in some cases. Access to facilities and offering intramurals may not be sufficient methods to increase physical activity among female youth, and strategies that promote community partnerships and supportive physical activity environments may be more effective [23]. The number of female varsity sports, partnerships with external fitness facilities to offer reduced memberships for students and the presence of a fitness ambassador were all positively and significantly associated with female physical activity. These specific programs and policies may foster motivation for physical activity through positive role modeling and a supportive physical activity environment [57,58,59].

Schools with greater numbers of female varsity sports had significantly higher percentages of females and males meeting the CSEP guidelines, higher average weekly days of strength training and higher average daily MVPA, all compared to schools with fewer varsity sports. Specific to the positive association with female physical activity outcomes, this finding is not surprising, as female varsity sports provide an opportunity for female physical activity, and opportunity for physical activity is an important predictor of physical activity [60,61,62]. Additionally, schools with more varsity sports opportunities may foster a more positive physical activity environment, encouraging higher physical activity among female and male youth [59]. Interestingly, offering a greater number of female-only and co-ed intramurals was not related to any female outcomes of physical activity. Other research has found similar results, as the availability and use of intramural and club activities were unrelated to student physical activity [23]. This may be explained by a difference in the demand of varsity sports compared to intramurals, where varsity sports are typically more intense and require more time commitment for practices and competition compared to intramurals, therefore contributing more physical activity [63]. There could also be differences in the physical activity environments in schools with higher numbers of intramurals, as these schools may be more focused on inclusion and enjoyment as opposed to competition and intensity, ultimately affecting the physical activity frequency, intensity and duration of the students’ MVPA.

Among female youth, access to external health and fitness facilities via reduced cost memberships was more important for physical activity compared to access to school facilities and equipment. This finding has been previously reported, as students were more physically active if they attended a school with established community partnerships such as those with community-based recreation facilities [23]. Physical activity enjoyment is positively associated with female physical activity [55] while competing priorities such as increased school work negatively associated with female physical activity [57,64]. These partnerships with fitness facilities could address both of these important factors by offering females an opportunity to participate in enjoyable physical activity on their own time, potentially explaining this positive finding [57]. Additionally, perceived competitiveness and feelings of intimidation are negatively associated with female physical activity, and these partnerships may provide alternative settings for female physical activity that are free of the competition and intimidation typical in group settings such as intramurals and physical education classes [22]. Additionally, interpersonal factors such as perceived parental and friend support for MVPA are positively associated with female physical activity [55]. Although we did not collect data on facility use, the utilization of external health and fitness facilities by parents and peers may encourage youth to utilize these facilities as well [55,57], and the reduced cost of access to such facilities may make this possible on a student budget. Lastly, previous research suggests that aesthetics and maintenance of indoor and outdoor facilities are important factors in the relationship between facilities and youth physical activity [29].

Schools with a fitness ambassador had significantly higher percentages of students meeting the CSEP guidelines, higher average weekly days of strength training and higher average daily MVPA, for female students. Female students may be more motivated to be physically active if they have a fitness ambassador, who is someone intended to provide support, education and motivation for physical activity participation on campus. Although no research was found evaluating the effectiveness of a fitness ambassador in secondary school specifically, there are studies suggesting that role models who support and encourage physical activity participation are positively associated with female physical activity [23,57,58]. Mothers, fathers and physical education teachers are all role models that support youth physical activity, and fitness ambassadors may act as similar role models by encouraging motivation and physical activity engagement [58]. Previous research has shown social connectedness to be important for the enjoyment of physical activity among female youth [44]. The presence of a fitness ambassador may provide this social connectedness, while also promoting feelings of support and connectedness to the school [21,23], all of which are important for physical activity among female youth. This finding is supported by other research suggesting that comprehensive school-based approaches to physical inactivity are most effective when they include community engagement, parental involvement and changes to the school environment [65,66,67].

### Limitations

Firstly, schools in COMPASS were recruited using convenience sampling, potentially limiting the generalizability of the results. However, COMPASS has a large sample size and uses active-information, passive-consent protocols [68], which helps limit self-selection and response biases and generates more robust results [50]. Secondly, this study was cross-sectional, so the directionality of the relationship between the school programs and policies and physical activity outcomes cannot be inferred. However, this study highlights the potential importance of varsity sports, fitness ambassadors and reduced external fitness memberships for female physical activity which should be examined longitudinally in future work to explore temporality. Thirdly, the data on student physical activity outcomes are self-reported, which may introduce self-reporting biases such as social desirability bias. However, the self-reported physical activity measures have adequate validity for use in school-level research [52]. Additionally, the associations examined were bivariate due to sample size constraints, and did not control for competing factors, potentially overestimating the true significance of the association in the presence of other factors. Lastly, the data examined physical activity outcomes at the school level, so the effect of these school-level programs and policies on youth physical activity cannot be extrapolated to the average student. However, examining associations at the school level is important for school-level policy making, as decision makers should consider implementing programs and policies that will have large reach and positively impact the health of the school population [47].

## 5. Conclusions

The results of this study highlight the differences in school-level female and male outcomes of physical activity. Additionally, this research suggests that some school-level programs and policies may be more effective at promoting physical activity among females. Specifically, varsity programs, reduced memberships to external fitness facilities and fitness ambassadors were all positively associated with female physical activity. Considering that female youth are an at-risk group for physical inactivity, implementing these school-level programs and policies may be effective methods to promote physical activity among females.

## Figures and Tables

**Table 1:** School-Level Physical Activity Outcomes of Male and Female Students from Year 7 (2018–2019) of the COMPASS Study.

Physical Activity Outcomes	Females		Males		Differences (Males-Females)		Paired *t*-Test
	Mean	SD	Mean	SD	Mean	SD	*p*-Value
Meeting CSEP Guidelines (%)	32.2%	9.2%	44.0%	10.0%	11.8%	6.0%	**<0.0001**
Days of Strength Training	2.2	0.4	2.7	0.4	0.5	0.3	**<0.0001**
Average Daily MVPA (min/day)	97.3	19.3	121.4	22.5	24.1	13.3	**<0.0001**

Values significant at α = 0.05 are bolded.

**Table 2:** Associations between School-Level Programs and Physical Activity Outcomes among Female Students from Year 7 (2018–2019) of the COMPASS Study.

School-Level Programs	Female MVPA (Minutes/Day)		Female Strength Training (Days)		Female Meeting CSEP Guidelines (%)	
Intramural and Varsity Sports:	Correlation Coeff.	*p*-Value	Correlation Coeff.	*p*-Value	Correlation Coeff.	*p*-Value
# Co-ed Intramurals	−0.16	0.0685	−0.05	0.5399	−0.08	0.3563
# Co-ed Individual Intramurals	0.02	0.8136	0.03	0.7579	0.05	0.5881
# Co-ed Team Intramurals	−0.26	0.0019	−0.11	0.2174	−0.17	0.0536
# Female Intramurals	0.06	0.5147	0.00	0.9937	−0.01	0.8975
# Female Individual Intramurals	−0.12	0.1526	−0.09	0.2719	−0.12	0.1768
# Female Team Intramurals	0.11	0.1938	0.03	0.6958	0.03	0.7352
# Female Varsity	0.60	**<0.0001**	0.37	**<0.0001**	0.50	**<0.0001**

Values significant at α = 0.05 are bolded.

**Table 3:** Associations between School-Level Policies and Physical Activity Outcomes among Female Students from Year 7 (2018–2019) of the COMPASS Study.

School-Level Policies		Female Meeting CSEP Guidelines (%)		Female Strength Training (Days)		Female MVPA (Minutes/Day)	
*Partnerships*							
		ANOVA		ANOVA		ANOVA	
Predictor Variables		Mean (sd)	*p*-Value	Mean (sd)	*p*-Value	Mean (sd)	*p*-Value
Access to external health and fitness facilities	No	31.7% (9.9%)	0.6121	2.22 (0.37)	0.7592	94.75 (18.61)	0.2195
	Yes	32.5% (8.7%)		2.21 (0.34)		98.91 (19.64)	
Reduced cost	No	31.4% (9.2%)	**0.0476**	2.20 (0.36)	0.2694	95.51 (19.30)	**0.0417**
	Yes	35.2% (8.5%)		2.28 (0.33)		103.71 (18.08)	
Fitness ambassador	No	31.0% (9.0%)	**0.0004**	2.18 (0.35)	**0.0062**	94.17 (18.83)	**<0.0001**
	Yes	38.4% (7.5%)		2.40 (0.31)		113.26 (12.70)	
***Access to School Facilities and Equipment during School Time***							
Access to indoor facilities during non-instructional times	No	38.4% (5.2%)	**0.0017**	2.38 (0.20)	**0.0266**	110.92 (9.30)	**0.0011**
	Yes	31.2% (9.3%)		2.19 (0.36)		95.18 (19.57)	
Access to outdoor facilities during non-instructional times	No	31.7% (12.1%)	0.8958	2.24 (0.37)	0.7926	100.31 (18.01)	0.5871
	Yes	32.1% (9.0%)		2.21 (0.35)		97.13 (19.45)	
Access to equipment during non-instructional time	sometimes/never	33.7% (9.2%)	**0.0178**	2.24 (0.37)	0.2191	100.98 (18.76)	**0.0051**
	Always	29.9% (8.7%)		2.17 (0.31)		91.61 (18.84)	
***Access to School Facilities and Equipment Outside of School Time***							
Access to indoor facilities outside of school hours	No	34.0% (10.4%)	0.2562	2.21 (0.41)	0.9415	105.61 (22.18)	**0.0135**
	Yes	31.7% (8.9%)		2.21 (0.34)		95.29 (18.08)	
Access to outdoor facilities outside of school hours	No	35.5% (9.1%)	0.0456	2.29 (0.33)	0.2558	103.58 (16.87)	0.0697
	Yes	31.4% (9.1%)		2.20 (0.36)		95.84 (19.57)	
Access to equipment outside of school hours	No	34.3% (9.1%)	**0.0218**	2.28 (0.34)	0.0598	101.24 (19.07)	**0.0468**
	Yes	30.7% (9.0%)		2.17 (0.35)		94.56 (19.06)	

Values significant at α = 0.05 are bolded.

## Data Availability

The datasets generated and analyzed for this study will not currently be shared because this is an ongoing study; however, access to the data supporting the findings of this study can be requested at https://uwaterloo.ca/compass-system/information-researchers (accessed on 22 March 2021).

## Appendix A

**Table A1:** Sociodemographic Characteristics of the School Sample (*n* = 136) from Y7 of the COMPASS Study.

Sociodemographic Characteristics		Sample	
		*n*	%
Total		136	100%
Province	AB	8	5.9%
	BC	15	11.0%
	ON	61	44.9%
	QC	52	38.2%
Urbanicity	Large Urban	58	42.6%
	Medium Urban	14	10.3%
	Small Urban/Rural	64	47.1%
Median Income	<$50,000	35	25.7%
	$50,000–75,000	33	24.3%
	$75,000–100,000	36	26.5%
	>$100,000	32	23.5%
School Size	≤300	30	22.1%
	301–500	35	25.7%
	501–950	55	40.4%
	>950	16	11.8%
